# Targeted Resequencing Reveals *ALK* Fusions in Non-Small Cell Lung Carcinomas Detected by FISH, Immunohistochemistry, and Real-Time RT-PCR: A Comparison of Four Methods

**DOI:** 10.1155/2013/757490

**Published:** 2013-01-20

**Authors:** Katja Tuononen, Virinder Kaur Sarhadi, Aino Wirtanen, Mikko Rönty, Kaisa Salmenkivi, Aija Knuuttila, Satu Remes, Aino I. Telaranta-Keerie, Stuart Bloor, Pekka Ellonen, Sakari Knuutila

**Affiliations:** ^1^Department of Pathology, Haartman Institute, University of Helsinki, P.O. Box 21 (Haartmaninkatu 3), 00014 Helsinki, Finland; ^2^Department of Pathology, HUSLAB, Helsinki University Central Hospital, P.O. Box 400, 00029 HUS, Finland; ^3^Division of Pulmonary Medicine, Department of Medicine, University of Helsinki and Helsinki University Central Hospital, P.O. Box 340, 00029 HUS, Finland; ^4^Lab21 Ltd, 184 Cambridge Science Park, Cambridge CB4 0GA, UK; ^5^Technology Centre, Institute for Molecular Medicine Finland (FIMM), University of Helsinki, P.O. Box 20, 00014 Helsinki, Finland

## Abstract

Anaplastic lymphoma receptor tyrosine kinase (*ALK*) gene rearrangements occur in a subgroup of non-small cell lung carcinomas (NSCLCs). The identification of these rearrangements is important for guiding treatment decisions. The aim of our study was to screen *ALK* gene fusions in NSCLCs and to compare the results detected by targeted resequencing with results detected by commonly used methods, including fluorescence in situ hybridization (FISH), immunohistochemistry (IHC), and real-time reverse transcription-PCR (RT-PCR). Furthermore, we aimed to ascertain the potential of targeted resequencing in detection of *ALK*-rearranged lung carcinomas. We assessed *ALK* fusion status for 95 formalin-fixed paraffin-embedded tumor tissue specimens from 87 patients with NSCLC by FISH and real-time RT-PCR, for 57 specimens from 56 patients by targeted resequencing, and for 14 specimens from 14 patients by IHC. All methods were performed successfully on formalin-fixed paraffin-embedded tumor tissue material. We detected *ALK* fusion in 5.7% (5 out of 87) of patients examined. The results obtained from resequencing correlated significantly with those from FISH, real-time RT-PCR, and IHC. Targeted resequencing proved to be a promising method for *ALK* gene fusion detection in NSCLC. Means to reduce the material and turnaround time required for analysis are, however, needed.

## 1. Introduction

Chromosomal rearrangements involving anaplastic lymphoma receptor tyrosine kinase (*ALK*), a member of receptor tyrosine kinase family, have been detected in various cancers [1]. *ALK* rearrangements create an oncogenic ALK tyrosine kinase that activates many downstream signaling pathways resulting in increased cell proliferation and survival [1]. In non-small cell lung carcinoma (NSCLC), the most common *ALK* rearrangement is a fusion of *ALK* gene and echinoderm microtubule-associated protein-like 4 (*EML4*) gene, formed as a result of a small inversion within the short arm of chromosome 2, where the genes are located [2]. EML4-ALK fusion protein serves as a therapeutic target for an ALK tyrosine kinase inhibitor, which has showed promising results when used in treating NSCLC patients carrying *ALK* rearrangement [3–6]. In addition to *EML4*, other *ALK* fusion partners have also been reported in lung cancer, including *TFG* [7], *KIF5B* [8], and *KLC1* [9].

*ALK* gene rearrangements can be screened by various methods, including commonly used fluorescence in situ hybridization (FISH), immunohistochemistry (IHC), and real-time reverse transcription-PCR (RT-PCR). Furthermore, next-generation sequencing technology holds a great potential as a new tool for cancer diagnostics [10]. Each of these methods has their own advantages and disadvantages. Hence, the optimal method to detect *ALK* fusions in NSCLC remains to be determined.

In this study, we screened *ALK* gene fusion status by FISH, IHC, real-time RT-PCR and targeted resequencing. Our aim was to compare the results obtained from these four methods in order to see how well they correlate with each other. We especially wanted to find out how well FISH, IHC, and real-time RT-PCR support the sequencing results. Moreover, we aimed to prove the suitability of targeted resequencing for testing of formalin-fixed paraffin-embedded (FFPE) tumor material. To our knowledge, this is the first paper comparing these four methods as means of *ALK* gene fusion identification.

## 2. Materials and Methods

### 2.1. Patients

Archived formalin-fixed paraffin-embedded tumor specimens were collected from 87 non-small cell lung carcinoma patients operated during 2005–2011 at the Hospital District of Helsinki and Uusimaa (HUS), Finland. Based on the WHO 2004 classification, 80 of the patients were diagnosed with adenocarcinoma, four with large cell carcinoma, one with squamous cell carcinoma, and two with unclassified NSCLC. Selection of the patients for the analyses was not random. The selected patients were mainly nonsmokers and those with adenocarcinomas. The *EGFR* gene mutation status was previously assessed for 57 patients, of which 9 carried *EGFR* mutation. The mean age of the patients was 63.7 years, ranging from 44 to 82 years. About half of the patients were male (*N* = 45) and half were female (*N* = 42). The tumor tissue percentage of the specimens was confirmed by a pathologist to be >20%.

For *ALK* fusion testing, we applied dual-color, break-apart FISH, real-time RT-PCR, immunohistochemistry, and targeted resequencing. Interpretation of the results was done in double-blind manner without knowing the results by other methods.

### 2.2. Fluorescence In Situ Hybridization

We performed fluorescence in situ hybridization on 95 FFPE tumor specimens from 87 patients (two separate samples from 8 of the patients), for which the tumor tissue sections (2.5 *μ*m) were fixed on microscope slides. Deparaffinization of the sections, slide pretreatment, protease pretreatment, hybridization with Vysis LSI ALK Dual Color Break Apart FISH probes, slide washing, and counterstaining by DAPI were done following the manufacturer's protocol (Abbott Molecular Inc., Des Plaines, IL, USA). Paraffin melting and hybridization were performed using StatSpin ThermoBrite Slide Processing System (Abbott Molecular Inc.). The results were viewed by fluorescence microscope. For each sample, the number of fused signals, single orange signals, and single green signals was counted in 50 nuclei. Cells containing at least one green and orange signal pair split apart by ≥2 signal diameters (pair-signal type fusion), or a single orange signal without corresponding green signal (single-signal type fusion), were considered to contain *ALK* gene rearrangement (positive). Fused or adjacent green and orange signals, or a single green signal (deletion of the corresponding orange signal), were an indication of a negative cell. Samples with ≥15% of positive cells were considered positive. 

### 2.3. Immunohistochemistry

Immunohistochemical stainings were done to 14 specimens from 14 patients using mouse monoclonal ALK antibody (clone 5A4, Novocastra, Newcastle, UK), 1 : 100 with OptiView DAB detection Kit (Ventana, Tucson, Arizona, USA), and CC1 buffer in BenchMark XT (Ventana, Tucson, Arizona, USA). Due to long permission procedures, ALK immunostaining was performed only for FISH positive and some negative cases.

### 2.4. Real-Time RT-PCR

We evaluated 95 FFPE tumour specimens from 87 patients for the presence of an *EML4-ALK* gene fusion using the AmoyDx *EML4-ALK *fusion gene detection kit (Amoy Diagnostics, Xiamen, China). Following xylene deparaffinization of a 16 *μ*M FFPE section, total RNA was extracted using the RNeasy FFPE Kit (Qiagen GmbH, Hilden, Germany) according to the manufacturer's protocol. RNA was quantified using the Qubit fluorometric quantitation system (Life Technologies, Carlsbad, CA, USA). 100–500 ng of the extracted RNA was used for reverse transcription and real-time PCR in each of the four reactions of the *EML4-ALK *fusion gene detection kit according to the manufacturer's protocol. Reaction 1 amplifies *EML4-ALK* variants 1, 2, 3a, and 3b, reaction 2 *EML4-ALK* variants 4 & 4′, reaction 3 *EML4-ALK* variants 5a, 5b, 5′, and 8, and reaction 4 the reference gene beta-actin. All assays were performed on an ABI7500 instrument (Applied Biosystems, Foster City, USA). Assay reactions achieving Ct values of ≤30 cycles were considered positive for one of the variants detected by that reaction mixture.

### 2.5. Next-Generation Sequencing

#### 2.5.1. DNA Isolation

DNA was isolated from FFPE NSCLC tissue specimens using QIAamp DNA Mini Kit (Qiagen GmbH, Hilden, Germany). The isolation was performed according to the manufacturer's instructions for genomic DNA purification from paraffin-embedded tissue material with following modifications: xylene was added twice and 100% ethanol was added three times for careful paraffin removal, samples were incubated at 90°C for 1 h after overnight cell lysis, Buffer AL was added normally but an incubation at 70°C for 10 min was not performed, and in the last step of the protocol, QIAamp Mini spin column loaded with water was incubated at room temperature for 5 min before first centrifugation, and at 50°C for 5 min before the final centrifugation to increase the DNA yield. DNA was eluted in 50 *μ*L of purified water. DNA concentration was measured by Qubit fluorometer (Life Technologies).

#### 2.5.2. Target Capture

We selected 192 genes associated with lung cancer for target enrichment. Baits were designed to capture all exons, 3′UTR, and 5′UTR regions of all genes, including *ALK* and *EML4* genes. Additional baits were designed from intronic region between exons 19 and 20 of *ALK* gene to capture all breakpoint variants of *ALK* fusion gene. Baits were designed with e-array (Agilent Technologies, Inc., Santa Clara, CA, USA) and RNA baits obtained from Agilent. A total of around 1 MB region, including 2676 target regions, was captured.

#### 2.5.3. Targeted Sequencing

2-3 *μ*g of DNA, isolated from 57 formalin-fixed paraffin-embedded tumor specimens from 56 patients, was fragmented, ligated to adapters, and enriched for target regions using Agilent's SureSelect in-solution target capture and enrichment protocol. Paired-end sequencing of the target-enriched libraries was performed on Illumina HISeq2000 sequencer (Illumina, Inc., San Diego, CA, USA).

#### 2.5.4. Primary Analysis

Data obtained from sequencing was processed with a variant-calling pipeline (VCP) developed at the Finnish Institute of Molecular Medicine (FIMM) [11]. Sequence reads were filtered for quality, paired-end reads aligned to the reference genome with the Burrows-Wheeler Aligner (BWA) [12], and duplicate fragments removed by rmdup algorithm. For variant calling, VCP utilized SAMtools' pileup [13]. Single nucleotide variation (SNV) calling and read end anomaly (REA) calling were based on FIMM's own developed algorithm. Detection of indels was performed with the Pindel [14], and Circos [15] was used for visualization of anomalously mapping paired-end (PE) reads. Results were visualized in the Integrative Genomics Viewer (IGV) [16].

For identifying *ALK* fusions from data, all exons and intronic region of *ALK* (chr2:29,446,208-29,448,431 in build GRCh37 of the human reference genome) were checked for anomalously mapped paired-end reads within this region.

## 3. Results

FISH and real-time RT-PCR were performed on all the 87 NSCLC cases, targeted resequencing on 56 cases, and IHC on 14 cases. Successful results were obtained from all specimens by each method.  Table 1 lists all ALK-positive cases detected by FISH, IHC, real-time RT-PCR, and targeted resequencing.

We detected *ALK* rearrangement by FISH in five patients and one replicate sample. Two of the positive cases had pair-signal type fusion and three of them had single-signal type fusion. We also detected one ALK fusion-positive borderline case with 20% of positive cells observed by first reader. After evaluations by two other readers, it was, however, considered negative (<15% of positive cells). No *ALK* fusion was observed in this case by real-time RT-PCR, targeted resequencing, or IHC.

Using real-time RT-PCR, we identified *EML4-ALK* fusion variants 1, 2, or 3a/b (without distinguishing between them) in five patients and one replicate sample.

Targeted resequencing revealed four patients and one replicate sample harboring *EML4-ALK* fusion (the fifth positive patient was not studied by targeted resequencing). All positive cases by targeted resequencing had a breakpoint in intronic region between exons 19 and 20 of *ALK*, and in intron 13 of *EML4*, corresponding the fusion variant 1 in real-time RT-PCR system.  Table 1 shows detailed breakpoints of the fusions in *ALK* gene.

**Figure 1:**
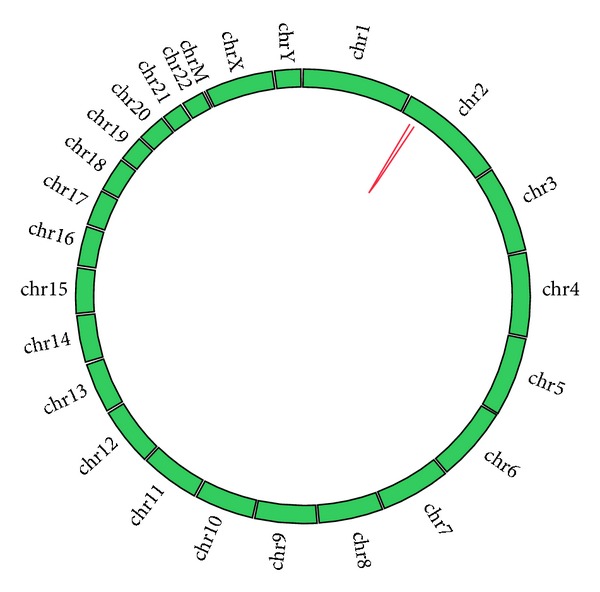
A Circos plot visualizing the detection of *EML4-ALK* fusion in lung cancer patient (LC14) by targeted resequencing.

**Figure 2:**
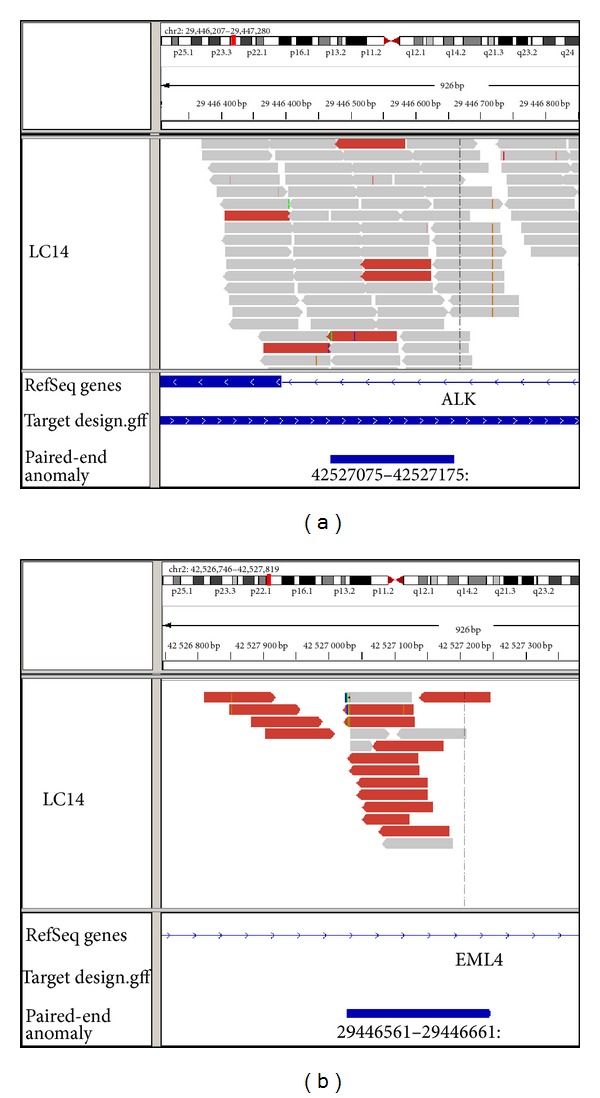
Detection of *EML4-ALK* fusion in lung cancer patient (LC14) by targeted resequencing. Targeted resequencing identified anomalously mapped pairs on chromosome 2. The pairs map to *ALK* (a) and *EML4* (b) in inverted orientation.

**Figure 3:**
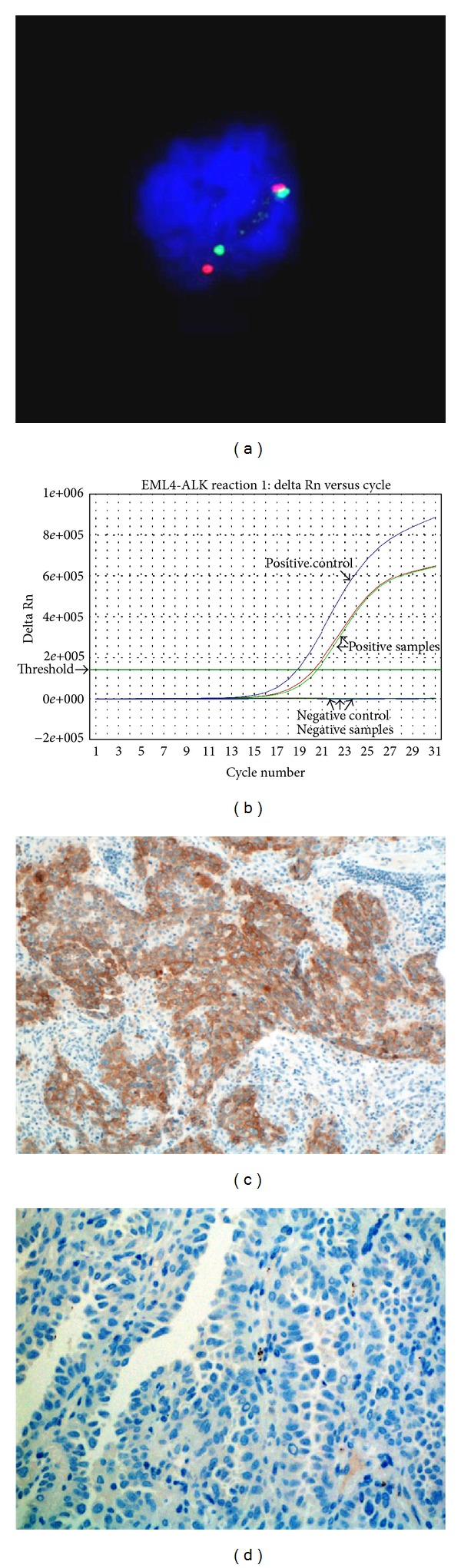
Detection of *ALK* fusion in lung cancer patient (LC14) by FISH, real-time RT-PCR, and immunohistochemistry. (a) FISH performed with Vysis LSI ALK Dual Color Break Apart FISH probes detected *ALK* fusion as split red and green signal. (b) An example results graph from real-time RT-PCR showing change in the normalized reporter signal (delta Rn) against PCR cycle number for reaction 1 of the AmoyDx *EML4-ALK* fusion gene detection kit. (c) and (d) Immunohistochemistry revealed strong expression of ALK in ALK-fusion positive patient (c) and no expression in ALK-fusion negative patient (d).

By IHC, we detected ALK staining in five cases. The results of FISH, real-time RT-PCR, targeted resequencing, and IHC corresponded to each other, and no discrepant results were observed. Figures 1 and 2 show a detection of ALK fusion-positive result by targeted resequencing, and Figure 3 represents examples of ALK fusion-positive results detected by FISH, real-time RT-PCR, and IHC. 

In total, we detected five (5.7%) ALK-positive cases in 87 NSCLC patients studied altogether. Three of these five *EML4-ALK* fusion containing tumors were adenocarcinomas and two were large cell carcinomas. Of all the studied cases, four men and one woman carried *ALK* fusion, while 41 men and 41 women were considered ALK negative. All the patients with *ALK* gene rearrangement were nonsmokers, and their age ranged between 44 and 63 years (mean age = 50.4 years). The age range among ALK fusion negative patients was 45–82 years (mean age = 64.5 years). None of the patients with *EGFR* mutation harbored *ALK *gene fusion. All replicate samples of the 8 patients included in our study showed concordant results in each method used.

## 4. Discussion

Non-small cell lung carcinoma patients harboring *ALK* gene rearrangement have been reported to benefit from tyrosine kinase inhibitor treatment [3–6], which makes the reliable screening of *ALK* fusions important. In addition to the commonly used FISH, IHC, and RT- PCR, targeted resequencing holds great potential in the detection of *ALK* rearrangements.

We studied NSCLCs using FISH, IHC, real-time RT-PCR, and targeted resequencing to detect *ALK* gene rearrangements. The results obtained from all four methods were interpretable and no discrepancies between the methods were observed.  Table 2 shows the comparison of the methods used for *ALK* fusion screening in this study. 

FISH is a relatively rapid, well-standardized, rather expensive method which only requires a small amount of tumor biopsy. The FISH assay performed in this study is the FDA-approved companion diagnostic for the NSCLC crizotinib, which uses probes that target to *ALK* locus on chromosome 2p23, thus recognizing all *ALK* gene rearrangements including non-*EML4-ALK* fusion partners. FISH could not, however, identify the fusion partner nor can it distinguish between different fusion variants. The interpretation of FISH results is rather subjective, and a considerable variability in interpretation of FISH results between readers have been reported [17]. In our study, all cases were evaluated by two experienced viewers, who read the signals for most of the cases fast (10 to 30 minutes) and reliably. The borderline ALK-positive case (20% of positive cells) observed by first FISH reading was evaluated by two other readers, who both detected <15% of positive cells. As the mean value of the three analyses was less than 15% of positive cells, the sample was considered, according to the provider of the FISH kit, negative, underlining the relevance of reevaluation of the borderline positive cases. The results from real-time RT-PCR, targeted resequencing, and IHC support the interpretation, since no *ALK* fusion was detected in this case by these methods. 

Immunohistochemistry is a rapid and relatively inexpensive method for diagnosing ALK-rearranged NSCLCs that require only a small biopsy sample. A great advantage of immunohistochemistry is that tissue morphology is retained during analysis, and thus the detection of *ALK* fusions can be focused on the tumor areas. We performed immunohistochemistry by using ALK antibody clone 5A4, the use of which has showed good sensitivity in *ALK* fusion detection in lung carcinomas [18–20]. In the present study, the results obtained from IHC were readily interpreted, and all ALK positive cases showed a strong positive staining. 

Real-time RT-PCR is a fast and sensitive method for detection of expressed *EML4-ALK* gene fusions in NSCLC. It recognizes known *EML4-ALK* fusion variants for which specific primers have been designed, not other *ALK* gene rearrangements. The real-time RT-PCR method used in this study was able to identify 10 *EML4-ALK* fusion variants of which variants 1, 2, or 3a/3b were detected. Only a small amount of RNA (100–500 ng) was needed for the test as a starting material. Although two samples gave only 84 ng and 90 ng of input RNA, the b-actin Ct values were considered acceptable (a Ct value <30). All in all, the assay worked 100% in all runs and the results were reproducible.

Targeted resequencing is a promising method for detection of *ALK* gene rearrangements. In the present study, the baits for sequencing were designed to capture all breakpoint variants of *ALK* fusion gene. We observed 73–96% of target 20-fold coverage in our samples, which shows a great efficiency of the target enrichment. All patients with *EML4-ALK* fusion detected by resequencing had *EML4-ALK* fusion variant 1, which is the most common variant observed in 33% of NSCLC patients [21]. The results obtained by targeted resequencing were interpretable and in significant correlation with results obtained by the commonly used methods, indicating the reliability and sensitivity of targeted resequencing in *EML4-ALK* gene fusion identification. However, an accurate sensitivity analysis by dilution series could not be performed using the material available for our study. Regarding the probability of false positive results, targeted resequencing did not detect any anomalously paired reads from *ALK* region in any of 51 *ALK* fusion negative (FISH, real-time RT-PCR, IHC) specimens, proving the specificity of targeted resequencing in *ALK* fusion detection. In addition to the detection of known *ALK* gene fusions, targeted resequencing holds a great advantage over other methods by enabling the simultaneous screening of novel *ALK* fusion partners as well as other lung cancer-related gene mutations, fusions and copy number alterations, saving time and material in the long run. In this study, we selected 192 lung cancer-related and miRNA genes for resequencing, although we only focused on the *ALK* fusions here. Targeted resequencing also provides a huge amount of novel information, for instance, increasing our knowledge of resistance of some NSCLCs to tyrosine kinase inhibitor treatment. However, the method still faces many challenges before it is applicable to an average laboratory of pathology. For example, expertise is needed for analysis and interpretation of the sequencing results, as well as means to lower the cost and speed up the turnaround time. Furthermore, sequencing requires a relatively large amount of DNA, which might be difficult to isolate from small FFPE biopsies. In the present study, the amount of DNA in two analyzed specimens was less than the optimal 2-3 *μ*g (1.6 *μ*g and 1.7 *μ*g, resp.) but interpretable results were obtained.

Similarly, as targeted resequencing can be used for detection of *ALK* gene rearrangements and other gene fusions in lung carcinomas, it can be applied on detection of fusions in other diseases, such as in hematologic malignancies and sarcomas, in which multiple chromosomal translocations have been characterized, some of them with clinical importance [22, 23].

We detected *ALK* gene fusion in 5.7% of NSCLCs, which supports previous studies reporting *EML4-ALK* fusion in ~3–13% of NSCLC patients [2, 3, 24–27]. However, our material does not present random cohort from NSCLCs, since the samples were mainly nonsmoking patients with adenocarcinoma. In our study, none of the patients with *EGFR* mutation had an *ALK* gene rearrangement (data not shown), which is in line with the literature, indicating that *EML4-ALK* fusion tends to occur in adenocarcinomas lacking *EGFR* mutation [17, 19, 26–29]. 

Formalin-fixed, paraffin-embedded tumor specimens are often the only tumor tissue material available for diagnostic purposes. Hence, the feasibility of FFPE to the detection methods is essential for reliable *ALK* fusion screening. In this study, we showed that FFPE material is applicable for all the four methods used for *ALK* gene fusion detection. However, it is important to note the low prevalence and limited selection of *ALK* gene fusions within the patient population, which is a common issue for the development, validation, and comparison of *ALK* gene fusion assays, as well as for the assessment of the clinical relevance of the various *ALK* gene rearrangements.

## 5. Conclusions

Our study proved that FISH, IHC, real-time RT-PCR, and targeted resequencing can be performed successfully on FFPE tumor specimen material. We found significant concordance among the four methods studied. Our results support the use and further development of targeted resequencing in detection of *ALK* gene fusions.

## Figures and Tables

**Table 1:** ALK positive cases detected by FISH, IHC, real-time RT-PCR, and targeted resequencing.

Sample ID	Age	Sex	Smoking	*FISH *	*IHC *	*RT-PCR *	*Targeted resequencing*
				Fusion type/% of positive cells		Variant type	Fusion/break position in ALK
LC1	46	F	Nonsmoker	Single-signal/70	Strongly positive	*EML4-ALK* variant 1/2/3a/3b	chr2: 29447508–29447608
LC11	48	M	Nonsmoker	Pair-signal/46	Strongly positive	*EML4-ALK* variant 1/2/3a/3b	chr2: 29446498–29446566
LC14*	44	M	Nonsmoker	Pair-signal/74	*Not studied *	*EML4-ALK* variant 1/2/3a/3b	chr2: 29446561–29446661
LC74*	44	M	Nonsmoker	Pair-signal/46	Strongly positive	*EML4-ALK* variant 1/2/3a/3b	chr2: 29446561–29446661
LC51	51	M	Nonsmoker	Single-signal/66	Strongly positive	*EML4-ALK* variant 1/2/3a/3b	chr2: 29446527–29446625
15915	63	M	Nonsmoker	Single-signal/40	Strongly positive	*EML4-ALK* variant 1/2/3a/3b	*Not studied *

*LC14 and LC74 are samples from the same patient but from a different FFPE block.

**Table 2:** Comparison of the methods used for *ALK* fusion detection in present study.

	FISH	IHC	Real-time RT-PCR	Targeted resequencing
Fusion types detectable	No fusion specification	No fusion specification	Only *EML4-ALK* fusions	All kind of fusions
Sensitivity	10–15%	5–10%	1–5%	Not determined
Time used for analysis	2-3 days	Half a day	1 day	10 days
Cost	Medium (~200 euros)	Low (~20 euros)	Medium (~150 euros)	High (~1000 euros)
Is FFPE material applicable?	Yes	Yes	Yes	Yes
Amount of material required	One tissue section (2.5 *μ*m thick)	One tissue section (2.5 *μ*m thick)	0.1–0.5 *μ*g of RNA	2-3 *μ*g of DNA
Possibility to see large range of other gene mutations in one analysis	No	No	No	Yes
The applicability to average laboratory of pathology	For most of the laboratories	For all laboratories	For some of the laboratories	Only for some laboratories
